# 

**Published:** 1890-10-18

**Authors:** 


### NOTICE TO CORRESPONDENTS.

All MS., Letters, Books for Review, and other matters intended for the Editor should be addressed The Editor, The Lodge, Porchester Square, London, W.

The Editor cannot undertake to return rejected M.S., even when accompanied by stamped directed envelope.

**Notice to Advertisers and Subscribers.**

All Advertisements, Orders for Copies of The Hospital, and Business Communications should be addressed to The Publisher, *not to the Editor*, The Hospital, 140, Strand, W.C.

**Bound Volumes of "The Hospital."**

Vol. VI., containing full page portraits of T.R.H. the Prince and Princess of Wales, and Miss Florence Nightingale, is obtainable at 4s., post free.

Vol. VII. is also for sale.

Orders will be received for Vol. VIII., 4s. post free. Cases for binding may be had of the Publisher, at 1s. 6d. each.

**To Residents, Secretaries, and Matrons.**

The Editor will be glad to have the Regulations and each Report as issued by Asylums, Hospitals, Convalescent and Nursing Institutions, as well as those of other Charities, and an early copy in duplicate of all Appeals and Festival Notices, etc., addressed to *The Editor, The Lodge, Porchester Square, London, W.* Any superintendent, secretary, matron, or other chief official who regularly sends such information may be registered, on application to the Publisher, to receive free of cost a cover for binding The Hospital in half-yearly volumes.

### BURDETT'S HOSPITAL ANNUAL FOR 1890

May be obtained of the Publisher, The Hospital Offices, 140, Strand, W.C., price 2s. 6d. limp boards, or 3s. 6d. cloth. All orders must be accompanied by a remittance.

### Scraps and Gleanings.

Dundee objects to paying £30,000 for a new hospital. We feel for the ratepayers.

Sir Sidney Waterlow is to be presented with his portrait, painted by Herkomer.

Dr. Stephen Mackenzie will deliver the Lettsomnian lectures in January next.

The Manchester Cremation Society has alloted £3,000 of shares, and is gaining ground in public favour.

Dr. James Cantlie has published an interesting account of his investigations into leprosy at Hong Kong.

The contractor for the supply of milk to Walton Workhouse has been fined £20 and costs for adulterating the milk with water.

Oban is not to have its cottage hospital after all; the owner of the proposed site objects to its being used for such a purpose.

Providence Free Hospital reports another year's successful work. The funds were materially increased last year by a grand bazaar.

The death of Mr. Clifford Winslow Turner is greatly regretted at Durham, Leeds, Newcastle, and other hospitals where he had worked.

Dr. Tidy has soothed the fears roused in Chester by Dr. Ballard's late report. Dr. Tidy says the death rate of Chester is decreasing, and the water is excellent.

Mr. Richard T. Holmes, hon. secretary, has issued a circular in favour of the Hospital Sunday collections, to be taken in Newcastle during November.

Great Northern Central Hospital.—The Committee have received a donation of £1,000 from Mr. W. H. Johnson for this poor, free, and unendowed charity.

An Irishman died at Whitehaven, it was said, of pneumonia, and a wake was held. Now twenty-one of those who attended the wake have typhus or typhoid fever.

Haydock Cottage Hospital issues a satisfactory report; during the year the interior has been painted and decorated. Sixty-two patients have been under treatment.

Miss Chalmers, of Cosmo Place, presented the small inmates of the Hospital for Sick Children with a large iced sponge-cake last Saturday, in honour of the harvest festival.

Professor Rawdon Macnamara presided at the annual dinner in connection with Meath Hospital, which was held on October 6th. Sir George Porter was among the speakers.

Sir James King presided at the twenty-first annual meeting of the Dunoon Convalescent Homes. Nearly 3,500 cases were received during the year; the report was most satisfactory.

Mr. Byramjee Jeejeebhoy died on September 12th. He was well known in India as a philanthropist, and he founded the medical schools at Ahmedabad and Poona, and built a hospital at Bombay.

The Hospital for Sick Children in Great Ormond-street held its harvest festival last Saturday. The lovely little chapel was exquisitely decorated, and the attendance both of inmates and outsiders was very large.

Typhoid is very prevalent in London just now, and a crying want is more accommodation for private patients. We offer the suggestion to a nurse with capital, social position, and influence, that it would pay well to open a private fever hospital.

The proposed fever hospital at Leytonstone has been protested against by 700 of the residents. Dr. Wilson urges a joint scheme between Leytonstone, Wanstead, Walthamstow, and Woodford; then they could select a site where the hospital would be a nuisance to no one.

Mr. Sheriff Augustus Harris and Mr. Sheriff Farmer attended the morning service last Sunday at St. Anne and St. Agnes, Gresham Street, when Canon Elwyn pleaded the cause of the Royal General Dispensary, Bartholomew Close. The collections at St. Olave's, Hart Street, last Sunday were for the Eastern Dispensary.

In order to benefit works of charity, including the Paris Urban Ambulance Society, the Minister of Finance has drawn up a scheme providing that the *Pari Mutuel* betting agencies be allowed to ply for business on the French racecourses, on condition that at least 2 per cent. of the sums be paid over to the Government.

For two years a girl has been suffering in the Cookham Union Workhouse Infirmary from what was supposed to be a diseased knee joint. The medical officer, when examining the knee a day or two ago, discovered the presence of a foreign substance imbedded in the knee, and after much difficulty succeeded in extricating a hairpin. How it came there and how long it had been in the knee is a mystery. The knee is now rapidly healing.

It will be interesting to see what support the proprietors of *Education* will receive from head masters for their scheme of establishing field classes for metropolitan and suburban schools. Anything which tends to draw a boy's attention away from the sordid surroundings of a town and to instil in him a love of nature, is worth the utmost encouragement. Nervous schoolmasters will probably object to boys filling their pockets with slow-worms and harmless snakes, especially if they crawl about the desks during school hours, and may prefer that their pupils should glean their knowledge of entomology and botany from books. We have no sympathy with them; books are necessary, but they will not accomplish much without nature as an aid.

### The Medical Students' Supplement.

[All communications for this Supplement should be addressed to The Editor of The Hospital, at 140, Strand, with the words "Students' Column" written in the left hand top corner of the envelope.]

### IN THE CAUSE OF MEDICAL STUDENTS.

The Editor of The Hospital has long had a desire to devote some columns of the paper to the interests of medical students, but there have always been difficulties in the way. The demands for The Hospital have, however, so much increased, that it has been determined to give up at least four columns each week entirely for the use of the students. In order to effect this, it has become necessary to increase and reorganise the pages of the paper. In the first place, information that is intended for people interested in hospitals and their working, apart from medical practitioners, will be transferred from November 1st to the Nursing Mirror supplement, which will be increased in size. By this arrangement it will be possible for The Hospital proper to be mainly devoted to medical and scientific topics without sacrificing any of the special features which have caused the paper to attain so wide a popularity.

In the Students' Supplement the Editor will endeavour to report from week to week the meetings of the various students' medical societies held at the different hospitals. He hopes that the secretaries of these societies will themselves send him regularly the minutes of their meetings, an abstract of the papers read, together with some notes on the discussion following the papers. It often happens that some very interesting papers are read at the students' medical societies which have hitherto, with the exception of one or two hospitals which print the proceedings of their societies themselves, been lost, for want of publication.

The Editor would also be pleased to receive the results of football matches and other athletic sports and games from the secretaries of the various clubs, together with any other Hospital news interesting to students generally.

### MEN OF THE FUTURE.

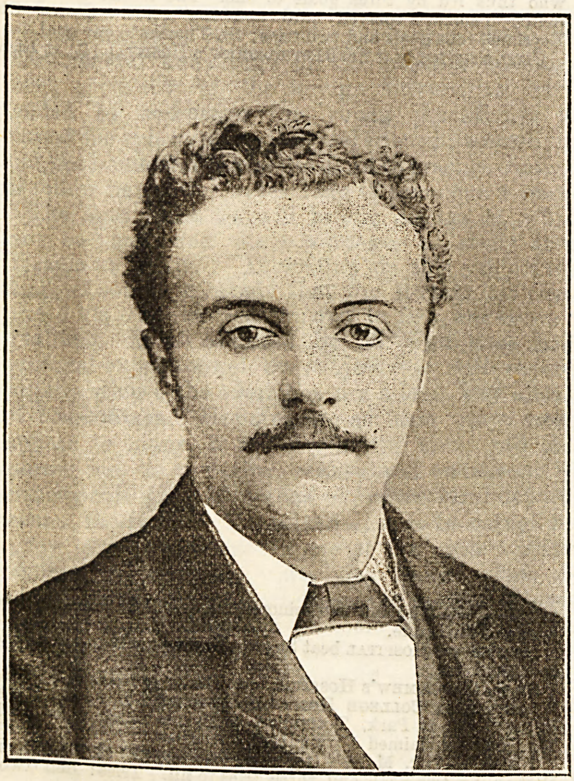

MR. T. CARWARDINE (Middlesex).
Gold Medalist and Exhibitioner, London University.

Mr. Thomas Carwardine, whose portrait we publish to-day, was educated at a public day-school, which he left at the age of fourteen, to enter a house of business in the City. He remained in this for five years. He then commenced to work for the London Matriculation Examination, which he passed in January, 1887, doing the greater part of the Preliminary Scientific examination in the following July. He entered as a student at the Middlesex Hospital in October, 1887, with an exhibition, and very soon passed the Primary Conjoint and then the Primary Fellowship Examination of the College of Surgeons. He was very successful in the hospital examinations, and was appointed junior demonstrator of anatomy to the medical school. In the recent Intermediate M.B. Examination of the London University, Mr. Carwardine obtained first class honours with the gold medal and exhibition in anatomy, and was high up in the honours list in physiology. So promising an early career should carry a man forward to even greater triumphs, and we wish Mr. Carwardine every success in the profession he is about to honour with his presence.

### STUDENTS' MEDICAL SOCIETIES.

### I.—University College Medical Society.

President—Mr. W. W. H. Tate. Hon. Secretaries—Mr. F. V. Bunch and Mr. W. H. B. Stoddart.

The opening meeting of this society was held in the Botanical Theatre, on Wednesday, October 8th. Mr. W. W. H. Tate, the president, was in the chair. The introductory address, entitled, "What? When? and How?" was given by Mr. Jonathan Hutchinson. The substance of this lecture we give in another part of our paper.

After Mr. Hutchinson's address (see page 44), Mr. W. W. H. Tate, the president, said: The society is proud of, and greatly honoured by, the presence of Mr. Jonathan Hutchinson, who had just given a lecture full of fresh news and of sound advice. He was sure all would join in giving three hearty cheers for Mr. Hutchinson.

Mr. Hutchinson then showed one or two specimens to illustrate more clearly what he meant by learning by the use of objects. He brought with him a model in the form of a raised map, especially designed, to show the distribution of leprosy in India. On this he demonstrated that, according to his theory, the distribution of leprosy was regulated by the consumption of excessive quantities of fish by the inhabitants. In the map the leprous areas were coloured black, and these occurred all round the coast line, and in the interior of the country in those areas which were shown on the map to be low lying, and hence the probability of the abundance of water and fish. He then exhibited two skulls which showed very clearly the areas of distribution of disease corresponding to the nerve supply of the part. Both of these skulls had areas of exostoses and hyperostoses in those regions supplied by the branches of the fifth nerve, and only on one side. One of these specimens was his own, the other belonged to Dr. Alexis Thompson, of Edinburgh.

The society then adjourned to the Flaxman Gallery, Anatomical Museum, and Mathematical Theatre, in which good histological specimens were shown to demonstrate different methods of staining and some pathological specimens of new growths. There was also a large assortment of some of the recent drugs and smaller instruments exhibited by Mr. W. Martindale.

### II.—St. Bartholomew's Hospital, Abernethian Society.

Presidents—Mr. Launcelot Andrews and Mr. E. J. Moore.
Vice-Presidents—Mr. H. M. Bowman and Mr. W. B. Addison.
Treasurer—Sir Wm. Savory, Bart., F.R.S.
Hon. Secretaries—Mr. G. B. Seton and Mr. A. F. Stevens.
Additional Committeemen—Mr. S. P. Cornish and Mr. L. Falkener.

The opening meeting of this society was held on Thursday, October 4th, in the Anatomical Theatre, the President (Mr. Launcelot Andrews) in the chair.

In opening the meeting the President stated that during the 95 years that the Abernethian Society had been in existence, the introductory address had not been given on a mental subject. He then added that only three years ago the introductory address was given by the much lamented Dr. Matthews Duncan, whose loss they were all now feeling

Dr. Claye Shaw, lecturer on psychological medicine to the hospital, then gave the introductory address. In commencing, he said a few appropriate words to the memory of the late Dr. Matthews Duncan, giving some interesting facts in connection with his life, which have not as yet been made public. He then passed on to the subject of the advance of civilisation, which was directly due to the progress of medical science. In the course of his address many valuable hints were given both to the students beginning their studies, and also to those who were well advanced with their curriculum. In conclusion, a few words were delivered on the founder of their society, named Abernethy. A vote of thanks to the lecturer was then proposed by Mr. Edgar Willett, and the meeting adjourned.

### III.—Guy's Hospital Pupils' Physical Society.

Honorary President.—Samuel Wilks, M.D., LL.D., F.R.S.

Vice-Presidents.—W. F. Colclough, B.A., A. E. Durham, B.A., M.B., B.C., H. E. Durham, B.A., J. Fawcett, J. M. Gill, M.B., F. W. Hall, H. V. Hickman, F. G. Hopkins, A. E. Norburn, A. T. Rake, A. J. Sharp, G. B. Smith, M.B., B.S., F. G. Swane Swayne, B.A., M.B., F. G. Vicars, C. F. Wakefield, and H. W. Webber.

Exhibits Committee.—W. F. Colclough, B.A., J. M. Gill, M.B., F. G. Hopkins, and C. F. Wakefield.

Hon. Secretaries.—Louis A. Dunn, M.S., and Lauriston E. Shaw, M.D.

The opening meeting of this society took place on Wednesday, October 1, at the Students' Club, the Honorary President, Dr. Samuel Wilks, being in the chair. The President first gave a hearty welcome, and some excellent advice to the large numbers of past and present students who were assembled.

Mr. G. A. Wright then gave the introductory address on "Medical Work in the North." He described at some length the development of the Manchester School of Medicine, which was founded in the year 1824 by Mr. Thomas Turner, and was then situated in Pine Street, at the back of the present infirmary. On the founding of the school, Dalton lectured on pharmaceutical chemistry, and Turner on anatomy, physiology, and pathology. In 1827 a second school was opened in Mount Street. After this, which was not a success, another one was started in Marsden Street. He said that almost all the provincial schools were modelled upon the Pine Street one, and were founded in the following chronological order: Sheffield, in 1827, organised by Overend and Thompson; Bristol, in 1828, by Hetling; Birmingham, in 1829, by Cox; Leeds, by Thackrah, and afterwards by Hey and Teale, in 1830; Norwich, also in 1830, by Crosse; Hull, in 1831, by Wallis; Nottingham and York, in 1833; and Gloucester, in 1834. After a vote of thanks for the instructive lecture, proposed to by Messrs. Norburn, Swayne, and Hickman, the Society adjourned.

### IV.—The Middlesex Hospital Medical Society.

President.—Mr. A. Pearce Gou d, F.R.C.S.
Vice-Presidents.—Mr. C. Gordon Brodie, F.R.C.S.; Dr. W. Essex Wynter.
Hon. Secretary.—Mr. T. T. Cockill.

The opening meeting of the Society was held on October 16th, when Mr. A. Lawson read a paper, entitled "That the proposed five years curriculum of medical study will be preferable to the present plan." Mr. Charles was opposed to the views of the reader of the paper.

There were exhibited at the meeting the prize histological specimens of last session, mounted by Mr. Kenneth Lawson, and also the prize pathological specimens mounted by Mr. Stanley.

### APPOINTMENTS.

[Early particulars of the higher appointments are invited for this column. Copies of letters of application and testimonials should be sent].

### St. Thomas's Hospital.

Appointed from Oct. 7th, 1890, to Jan 6th, 1891.

Clinical Clerks.

Dr. Bristow—Ellis, R. K., Harris, J. D., Simpson, H., Wright, S. F., Wyman, C., Wysard, A. T.

Dr. Ord.—Burden, H., Darker, G. F. J., England, H., Fawssett, R., Gillam, J. B.

Dr. Harley.—Patch, H. H. L., Spilsbury, F. H., Tribe, A. G., Watson, E. Y., Willock, T. H.

Dr. Payne.—Bensusan, A. D., Haines, A., Jepson, V. B., Renny, E. G.

Dr. Sharkey.—Frederick, H. J., Gabbett, P. C., Gorrall, J. G., Prall, C. B., Williams, G. C. W.

Dr. Cullingworth.—How, A., Milton, W. T. E., Morris, E. H. G., Round, J. C.

Clinical Dressers.

Mr. Sydney Jones.—Abel, H. M., Box, C. R., Fisher, J. H., Wainwright, W. G., Wallace, C.S., Walker, W. W.

Mr. Croft.—Campbell, A. M., Daniel, R. N., Isaacs, E. P., Purvis, W. P., Turner, J. G., Watkins, Pitchford W., Way, J. H. F., Williams, H. G.

Sir William MacCormac.—Banks, A., Parsey, E. W., Potter, W., Rock, C. H., Staddon, H. C., Ware, H. S.

Mr. MacKellar.—Hardwick, H. G. C., Harper, W., Jaffé, C. S., Jones, E. J. J., Law, R. R., Romer, H.

### SCHOLARSHIPS AND PRIZES.

### University College, Liverpool.

Holt Tutorial Scholars, A. J. Chalmers and W. E. Livesey. Lyon Jones Scholars, H. E. Annett, jun., and F. J. Woods, sen. Derby Exhibitioner, J. C. M. Given. Oakshott Prize, J. C. M. Given and V. B. Bennett (equal). Winter Session, 1889-90—Third and Fourth Year Subjects—Medicine, J. C. M. Given, Silver Medal, V. B. Bennett, and C. D. Holmes; Pathology, J. C. M. Given, Silver Medal; Obstretrics, G. Oldershaw, Silver Medal; Chemistry, J. A. Eatoch, Silver Medal. Second Year Subjects—Advanced Anatomy and Physiology, F. J. Woods, Gold Medal, and H. B. Dickinson, Bronze Medal; Histology Prize, C. E. Lister and J. P. Nixon (equal). Summer Session, 1890—Materia Medica, J. A. Eatock and W. H. Budd; Forensic Medicine, V. B. Bennett, Silver Medal; Practical Medicine, H. G. Harding, Silver Medal. Students' Debating Society—First Prize for Essay, A. I. Thomas; First Prize for Debating, J. Evans; Prize for Medical Cases, D. F. Williams.

### ATHLETICS.

### Football.

The following matches were played on October 11th:—

St. Thomas's Hospital v. Old Leysians.—This match was played at Stamford Bridge, in most delightful weather. The Old Leysians kicked off, Stonar picked up the ball, off side was claimed and allowed, and Gould placed a goal from a free kick. Then Whitworth (Leysians) got a try, and Gould kicked a goal. After this Ashford (Thomas's) kicked a goal. Just before half time Gould succeeded in kicking another goal for the Leysians, who thus led by three goals to one. The second half of the match Thomas's played hard a losing game. Scores—Old Leysians, four goals and two tries; St. Thomas's, one goal.

Civil Service beat St. Bartholomew's Hospital (R) at Richmond by two tries and two minors to one minor. Tries by Freeman and Brownlie.

Middlesex Hospital beat Sutton by two goals and four tries to a goal.

St. Bartholomew's Hospital beat Foxes by four goals to one.

University College Hospital drew with London Welsh (second) (R) at Park, Tottenham, with one minor point each. The Hospital claimed a try gained after time.

Wasps beat St. Mary's Hospital (second) (R) at Half Moon, Putney, by two goals and three tries to nil. Tries: Bell (two), Soor, Poore, and Spiller.

### EVENTS FOR THE MONTH OF OCTOBER.

[Items intended for this column must reach the office, 140, Strand, W.C., not later than first post on Tuesday mornings.]

### Medical Societies.

St. Bartholomew's Hospital, Abernethian Society.—October 23, Mr. A. A. Kantack, F.R.C.S., "On Inflammation and Repair"; October 30, Mr. D'Arcy Power, F.R.C.S., "The Cure of the King's Evil."

Guy's Hospital Physical Society.—October 18, Mr. J. B. Leathes, B.A., "Tobacco."

St. Mary's Hospital Medical Society.—October 22, Mr. H. Davis, "Anæsthetics"; Mr. W. J. Foster, "Clinical Cases."

### Athletics.

Football Matches.—St. Thomas' Hospital first fifteen: October 18, v. Blackheath, at Blackheath; October 29, v. Cambridge, at Cambridge. St. Thomas' Hospital, second fifteen: October 18, v. London Welsh second fifteen, at Balham; October 25, v. Crusaders, at Ealing.

